# The Status and Influencing Factors of Cyberchondria During the COVID-19 Epidemic. A Cross-Sectional Study in Nanyang City of China

**DOI:** 10.3389/fpsyg.2021.712703

**Published:** 2021-11-11

**Authors:** Xiao-Qing Peng, Yang Chen, Yi-Chuan Zhang, Fei Liu, Hai-Yan He, Ting Luo, Ping-Ping Dai, Wen-Zhao Xie, Ai-Jing Luo

**Affiliations:** ^1^The Third Xiangya Hospital, Central South University, Changsha, China; ^2^Xiangya School of Public Health, Central South University, Changsha, China; ^3^Key Laboratory of Medical Information Research, Central South University, College of Hunan Province, Changsha, China; ^4^School of Life Sciences, Central South University, Changsha, China; ^5^The Second Xiangya Hospital of Central South University, Changsha, China

**Keywords:** cyberchondria, health anxiety, health-related information seeking, residents, COVID-19

## Abstract

Cyberchondria is considered “the anxiety-amplifying effects of online health-related searches.” During the COVID-19 pandemic, people are likely to search health-related information online for reassurance because of fear and related physical symptoms, while cyberchondria may be triggered due to the escalation of health anxiety, different online seeking behavior preference, information overload, and insufficient e-health literacy. This study aimed to investigate the status and influencing factors of cyberchondria in residents in China during the epidemic period of COVID-19. The participants were 674 community residents of Nanyang city surveyed from February 1 to 15, 2020. We administered online measures, including the Chinese Short Form of the Cyberchondria Severity Scale (C-CSS-12), Short Health Anxiety Inventory (SHAI), eHealth Literacy Scale (eHEALS), Patient Health Questionnaire-15 (PHQ-15), and COVID-19-related online information seeking behavior questionnaire. In our study, the average C-CSS-12 total score of residents was 30.65 ± 11.53 during the virus epidemic; 25% of participants scored 22 or below, 50% scored 23 to 38, and 21.9% scored 39 to 60. The SHAI total score (β = 0.598 > 0, *P* < 0.001), the use of general search engines (β = 1.867 > 0, *P* = 0.039), and searching for information on how to diagnose COVID-19 (β = 2.280 > 0, *P* = 0.020) were independent risk factors for cyberchondria, while searching lasting less than 10 min each (β = −2.992 < 0, *P* = 0.048), the use of traditional media digital platforms (β = −1.650 < 0, *P* = 0.024) and professional medical communication platforms (β = −4.189 < 0, *P* = 0.007) were independent protective factors. Our findings showed that nearly a quarter of the participants scored 39 or higher on the C-CSS-12 in Nanyang city during the pandemic, which should be taken seriously. Health anxiety and COVID-19-related online information seeking behavior including online duration, topics and choice on different information channels were important influencing factors of cyberchondria. These findings have implications for further research and clinical practice on cyberchondria in China.

## Introduction

In recent years, health-related internet usage has grown rapidly. By June 2019, there were 4.5 billion internet users worldwide, with the majority located in Asia (50.7%), followed by Europe (16%) and Africa (11.5%) (Vismara et al., 2020). And an American survey showed that 88% of American internet users searched for medical information online (Vismara et al., 2020). According to the 48th “Statistical Reports on Internet Development in China” (China Internet Network Information Center, 2021), by June 2021, China’s Internet users has reached 1011 million, and according to the “2018 Chinese Internet users’ popular science demand search behavior report” (China Association for Science and Technology, 2018), the proportion of health and medical science inquiries accounted for 66.83% of the total, indicating that health information has been accessible via the internet to an increasing number of people hoping to better understand their own health and to obtain reasonable explanations for relevant symptoms. However, online health information searches have the potential to escalate medical concerns (Navas-Martin et al., 2012) and trigger unnecessary worry about health. This phenomenon is referred to as “cyberchondria” (Loos, 2013).

Cyberchondria is an “emerging risk” accompanied by the information era. Since cyberchondria was proposed by news media in the mid-1990s and coined from a combination of “cyber” and “hypochondriasis” (Loos, 2013; Vismara et al., 2020), it has received some attention from researchers in recent years. Some researchers have proposed that it is considered as “the anxiety-amplifying effects of online health-related searches” (Starcevic, 2017) and denotes repeated and excessive online searches for health-related information that are associated with increasingly higher levels of health anxiety than before the search (Baumgartner and Hartmann, 2011; Muse et al., 2012). Others have argued that cyberchondria is a “multi-dimensional structure,” including excessive and repeated online health information searches, negative emotional states related to online health information searches, the resulting interruption of other activities and doctor consultations due to increased anxiety (McElroy and Shevlin, 2014).

Cyberchondria is closely related to health anxiety and online health information searches (Starcevic, 2017). A group of studies (Baumgartner and Hartmann, 2011; Muse et al., 2012; Fergus, 2013; Fergus and Dolan, 2014; Doherty-Torstrick et al., 2016; McMullan et al., 2019; Vismara et al., 2020) found that there is a moderate to strong relationship between health anxiety and cyberchondria. Subjects with elevated health anxiety suffer greater anxiety during and after online health-related searches. Even individuals with low levels of health anxiety may experience increased anxiety when searching online (Tyrer, 2018; Tyrer et al., 2019). When people browse the internet for their common and possibly harmless symptoms, they tend to escalate to look for more serious and rare symptoms. This escalation may be related to the way information is presented such as ranking, terminology, and the user’s preference for more serious explanations of the illness (White and Horvitz, 2009), which may lead to more frequent and longer searches. Studies have shown that searching for health information may indeed increase levels of distress and uncertainty about one’s feared condition (White and Horvitz, 2009; Baumgartner and Hartmann, 2011; Doherty-Torstrick et al., 2016) and that there is a positive correlation between health anxiety and online health-related information seeking frequency and duration (McMullan et al., 2019). Due to the ambiguity of online health information (Eysenbach et al., 2002; McMullan et al., 2019), difficulties in filtering and acquiring clear information is a key anxiety-amplifying factor related to cyberchondria (Starcevic, 2017). Individuals seeking reassurance about their health may spend much of their time attempting to determine the validity of health-related information, and this process contributes to the cycle in which repeated online searches increase distress and anxiety (Starcevic and Berle, 2013). Some scholars have proposed that e-health literacy could negatively moderate the indirect effect of affective responses on cyberchondria (Zheng et al., 2020) and that improving it may be an effective intervention for cyberchondria.

Other studies reported that problematic internet use (PIU) appears highly relevant to cyberchondria (Starcevic and Berle, 2013; Fergus and Spada, 2017). Besides, intolerance of uncertainty (IU) and anxiety sensitivity (AS) may confer vulnerability for cyberchondria (Norr et al., 2015a), and the relationship between health anxiety and the frequency of internet searches for medical information grows increasingly stronger as IU increased (Fergus, 2013). Additionally, obsessive-compulsive symptoms, especially for contamination/washing and responsibility for harm symptoms, are positively correlated with cyberchondria (Norr et al., 2015c). For the consequences of cyberchondria, studies have revealed that it is associated with negative health outcomes such as functional impairment, lower quality of life, less satisfaction with doctor consultation, increased health care utilization (Barke et al., 2016; Doherty-Torstrick et al., 2016; Tanis et al., 2016; Mathes et al., 2018).

As we all know, the COVID-19 pandemic is a global crisis that causes high morbidity and mortality and has been declared by the World Health Organization (WHO) to be a public health emergency (Jungmann and Witthoft, 2020). During the pandemic, due to movement restriction issued by governments and social isolation measures, many Chinese people rely on the internet for COVID-19-related health information (Huang and Zhao, 2020) to better understand the disease and spent more time on it to seek reassurance (Jacobs et al., 2017; Jungmann and Witthoft, 2020; Kiraly et al., 2020; Wang et al., 2020; Zhao et al., 2020). However, the vast amount of information can be confusing (Navas-Martin et al., 2012), especially when the reliability and credibility of information provided by different information sources and channels varies (Cui et al., 2020; Gehrau et al., 2021). Moreover, the novel coronavirus has caused a widespread search for information with the dissemination of unregulated or misleading health information (Cuan-Baltazar et al., 2020; Song et al., 2021). Both of them potentially result in overconcern even anxiety (Doherty-Torstrick et al., 2016; Jungmann and Witthoft, 2020). Recent studies have showed that excessive media consumption and information overload during the COVID-19 pandemic is associated with increased anxiety (Farooq et al., 2020; Gao et al., 2020; Laato et al., 2020) and that virus anxiety (Jungmann and Witthoft, 2020) and fear of COVID-19 (Seyed Hashemi et al., 2020) is positively correlated with cyberchondria. Starcevic V pointed that the factors that contribute to cyberchondria at this time include a heightened perception of threat and fear of a newly identified and poorly understood disease, lack of authoritative and trustworthy sources of relevant health information, difficulty in coping with abundance of information that is often confusing, conflicting, unverified and constantly updated, along with a decreased ability to filter out unnecessary information and inability of excessive online health information seeking to provide the necessary information and deliver reassurance and so on (Starcevic et al., 2021).

Accordingly, during the pandemic, people are likely to search health-related information online because of fear of COVID-19 and related physical symptoms, while cyberchondria may be triggered due to the escalation of health anxiety, different online seeking behavior preference (such as frequency, duration, topics, choice on information channels), information overload, and insufficient e-health literacy. However, research on cyberchondria is still in its infancy, and data on the status and influencing variables of cyberchondria are still scarce (Vismara et al., 2020),especially in residents in China during the epidemic period of COVID-19. This study aimed to understand the status of cyberchondria in residents during the pandemic and explore whether health anxiety, online information seeking behavior, e-health literacy, and physical symptoms have an impact on cyberchondria.

## Materials and Methods

### Participants and Procedure

**FIGURE 1:**
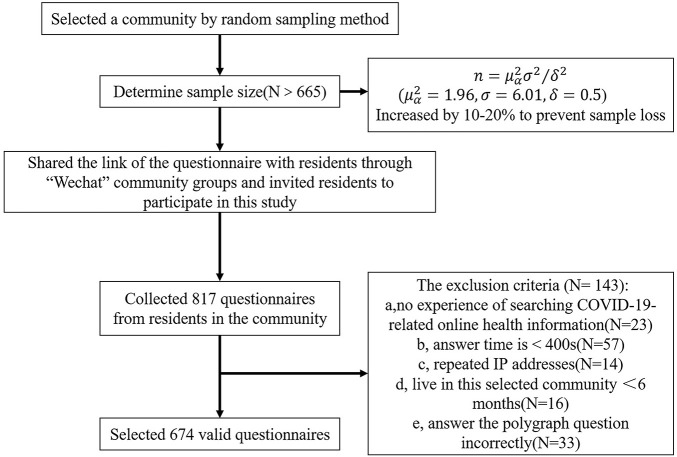
The process of participants sampling and recruitment.

The objects of this study are residents of a community in Nanyang city (located in central China, Henan Province), specifically residents who have lived in this area for more than 6 months. We selected a community by random sampling method. Referring to the standard deviation of CSS-12 score in a literature is 6.01 (Zheng et al., 2021), the required sample size was calculated (Fang, 2010; Sun and Xu, 2014) and increased by 10–20% to prevent sample loss. It was concluded that more than 665 samples should be selected to meet the demand. The questionnaire was made and uploaded to the popular online professional survey platform what is named “Wenjuanxing^1^” for data collection questionnaire surveys. Then we got permission from the community office, shared the link of the questionnaire with residents through “WeChat” community groups. WeChat has location-based online communities, and we arranged for WeChat community moderators to invite residents to participate in this study. The anonymous survey was conducted from February 1st to 15th, 2020. The inclusion criteria were as follows: (a) voluntarily participating in this study; (b) being able to understand and complete the questionnaire independently; (c) experience searching COVID-19-related online health information. The exclusion criteria were as follows: (a) no experience of searching COVID-19-related online health information; (b) answer time is <400 s (lower than the normal answer time); (c) repeated IP addresses; (d) live in this selected community for less than 6 months; (e) answer the polygraph question incorrectly. Interested participants were presented an online informed consent statement and each participant was compensated with 1–3 CNY for his or her participation. All procedures were approved by the institutional review board (IRB) of the Third Xiangya Hospital, Central South University. There were 817 questionnaires from community residents who completed the survey. However, 143 questionnaires were removed due to the exclusion criteria, and 674 valid questionnaires were selected (82.5%). The process of participants sampling and recruitment in Figure 1.

### Measures

#### Chinese Short Form of the Cyberchondria Severity Scale

McElroy and Shevlin (2014) developed the first trial version of the Cyberchondria Severity Scale (CSS) dedicated to assessing the severity of cyberchondria. The CSS has a total of 33 items and includes five subscales: Compulsion, Distress, Excessiveness, Reassurance Seeking and Mistrust of Medical Professional. McElroy et al. (2019) revised it into the 12-item Cyberchondria Severity Scale Short Form (CSS-12) and deleted “Mistrust of Medical Professional” because several authors considered this subscale to be distinct from cyberchondria and strongly recommended its removal (Fergus, 2014; Norr et al., 2015b).

The C-CSS-12 was translated from the CSS-12 and semantically adapted for the Chinese population. It has the same total items as the CSS-12 and uses a five-point Likert-type scale ranging from “never” to “always,” with a score of 1–5 points for each item and 60 points total. The higher the score, the higher the severity of the suspected cyberchondria (McElroy et al., 2019). The descriptions of CSS-12 items 5, 11, and 12 were modified during the process of translation. “General practitioners (GPs)” in item 5 of the original scale was changed to “community primary care physicians.” “My GP/medical professional” in item 11 and “consult with other medical specialists, e.g., consultants” in item 12 were simplified and changed to “physician” and “I might consult a specialist,” respectively. According to our studies on the reliability and validity of the C-CSS-12, the Cronbach’s α coefficient of the C-CSS-12 was 0.931, which matched the Cronbach’s α of 0.90 in the original version (McElroy et al., 2019), indicating a high level of internal consistency. In this study, the scale was used to measure the general cyberchondria under the virus epidemic.

#### Short Health Anxiety Inventory

The SHAI is an 18-item self-report questionnaire measuring health anxiety that includes two subscales: illness likelihood, IK (items 1–14) and negative consequence, NC (items 15–18). Items are rated on a four-point Likert-type scale ranging from 0 to 3, with a total score of 0–54. A total SHAI score of ≥15 points indicates health anxiety (Bailey and Wells, 2015); the higher the score, the higher the degree of health anxiety (Alberts et al., 2013). The Chinese version of the SHAI has good reliability and validity (Yuan et al., 2015). In this study, the internal consistency (Cronbach’s α coefficient) of the SHAI was 0.927. In this study, the scale was used to measure the general health anxiety under the virus epidemic.

#### EHealth Literacy Scale

The eHEALS is an 8-item self-report questionnaire assessing users’ combined knowledge, comfort, and perceived skills at finding, evaluating, and applying electronic health information to health problems. Items are rated on a five-point Likert-type scale ranging from 1 to 5, with higher scores indicating greater literacy. Both the original and Chinese versions of the scale have sufficient reliability and validity (Norman and Skinner, 2006; Sudbury-Riley et al., 2017; Wong and Cheung, 2019). In this study, the internal consistency (Cronbach’s α coefficient) of the eHEALS was 0.924.

#### Patient Health Questionnaire-15

The PHQ-15 mainly evaluates the degree of difficulty caused by various common physical symptoms in the past 4 weeks (Kroenke et al., 2002). It is an independent self-rating scale for somatic symptom groups and consists of 15 items, with a score of 0–2 points for each item and 30 points total. The higher the score, the more severe the physical symptoms. Scores of 0–4 are classified as no physical symptoms, 5–9 are classified as mild physical symptoms, 10–14 are classified as moderate physical symptoms, and 15–30 are classified as severe physical symptoms (Elhai et al., 2020). It has good internal consistency (Kroenke et al., 2002), criterion validity, and test–retest reliability (van Ravesteijn et al., 2009). The internal consistency in this study was 0.904.

#### General Questionnaire

The main content includes demographic information such as gender, age, occupation, education level, monthly income, self-report personal medical condition and relatives’ medical condition.

#### COVID-19-Related Online Information Seeking Behavior Questionnaire

COVID-19-related online information seeking behavior mainly includes active seeking frequency, duration, topics, and choice on different information channels. This questionnaire involves a series of questions associated with it.

#### Quality Control

We devised a common-sense question in the questionnaire for polygraph detection (what day is the National Day of China?) to filter out questionnaires with low authenticity. Besides, the questionnaires with repeated IP addresses and too short answer time were eliminated. Additionally, we checked the logic and completeness of the collected questionnaires.

### Statistical Analysis

Statistical analyses were performed with SPSS for Windows, version 25.0 (SPSS Inc., Chicago, IL, United States). Categorical variables were expressed as (%), and continuous variables were presented as mean (M) ± standard deviation (SD). Use percentile to describe the distribution of C-CSS-12 scores. The *t*-test or analysis of variance (ANOVA) was used to compare normally distributed continuous variables between two or more groups. The correlations among cyberchondria (C-CSS-12), health anxiety (SHAI), e-health literacy (eHEALS) and Patient Health Questionnaire-15 (PHQ-15) were verified using Pearson correlation. The independent factors of cyberchondria were determined using linear regression models. All probabilities were two-tailed, and the level of significance was set at 0.05.

## Results

### Participant Sociodemographic and Health Information

The sociodemographic and health information characteristics of participants are displayed in Table 1. Of the 674 respondents, approximately 43.6% (294/674) were male and 56.4% (380/674) were female. The participants’ age ranged from 16 to 70 years (average age: 32.67 ± 11.21) and were mainly distributed between 20 and 30 (33.7%, 227/674) and 31 and 40 (25.8%, 174/674) years old. The education level was mainly distributed at university/college degree or below (91.5%, 617/674). The respondents spanned all occupation ranks. The individual monthly income was mainly distributed at ≤4,500 CNY (79.4%, 535/674). The top four personal illnesses were chronic gastritis or peptic ulcer (7.9%), hypertension (7.4%), chronic bronchitis (5.0%), and HP infection (4.2%); the top four relatives’ illnesses were hypertension (28.5%), diabetes (16.8%), coronary heart disease (10.5%), and chronic bronchitis (6.2%).

**TABLE 1:** Sociodemographic and health information characteristics of participants.

Characteristic	Values (*N* = 674)
**Gender *n* (%)**	
Male	294 (43.6)
Female	380 (56.4)
**Age (years) *n* (%)**	
<20	108 (16)
20–30	227 (33.7)
31–40	174 (25.8)
41–50	110 (16.3)
>50	55 (8.2)
**Education level *n* (%)**	
≤High middle school	235 (34.9)
Undergraduate/college	382 (56.7)
≥Postgraduate	57 (8.5)
**Vocation *n* (%)**	
Civil servants	39 (5.8)
Educational practitioners	115 (17.1)
Medical practitioners	45 (6.7)
Media/IT practitioners	8 (1.2)
Practitioners of public security and law	5 (0.7)
Business managers	65 (9.6)
Professional skilled workers	60 (8.9)
Self-employed persons	34 (5.0)
Freelancers	86 (12.8)
Farmers	27 (4.0)
Students	131 (19.4)
Unemployed	27 (4.0)
Others	32 (4.7)
**Monthly income (CNY) *n* (%)**	
<2000	197 (29.2)
2000–3000	178 (26.4)
3001–4500	160 (23.7)
4501–6000	77 (11.4)
6001–8000	26 (3.9)
>8000	36 (5.3)
**Personal illness (top 4) *n* (%)**	
Chronic gastritis or peptic ulcer	53 (7.9)
Hypertension	50 (7.4)
Chronic bronchitis	34 (5.0)
Helicobacter pylori infection	28 (4.2)
**Relatives’ medical illness (top 4) *n* (%)**	
Hypertension	192 (28.5)
Diabetes	113 (16.8)
Coronary heart disease	71 (10.5)
Chronic bronchitis	42 (6.2)

### Distribution of Chinese Short Form of the Cyberchondria Severity Scale Scores of Participants

The C-CSS-12 total scores of participants ranged from 12 to 60. The average of the C-CSS-12 total scores was 30.65 ± 11.53, while that of “Excessiveness” subscale was 8.89 ± 3.25, that of “Compulsion” subscale was 6.80 ± 3.23, that of “Distress” subscale was 7.02 ± 3.42, and that of “Reassurance Seeking” subscale was 7.94 ± 3.27. It showed that 25% (172/674) of participants scored 22 or below, 50% (353/674) scored 23 to 38, and 21.9% (149/674) scored 39 to 60, which roughly reflected the severity level of cyberchondria (Table 2).

**TABLE 2:** The distribution of C-CSS-12 scores of participants (*n* = 674).

Categories	Corresponding raw score of C-CSS-12	*N* (%)
At or below 25th percentile	22 and below low	172 (25.5)
Above 25th and below 75th	Between 23 and 38	353 (52.6)
At or above 75th percentile	39 and above	149 (21.9)

### Characteristics of COVID-19-Related Online Information Seeking Behavior

The characteristics of COVID-19-related online information seeking behavior are displayed in Table 3. We found that 49.7% (335/674) of participants searched for COVID-related online information 1–3 times a day, 17.2% (116/674) searched 2–6 times a week, and 13.9% (94/674) searched six times a day or more. During the online search for COVID-19-related information, nearly half of the participants (48.4%, 326/674) searched for 10 min to 30 min each time. With regard to the choice on different information channels, the most commonly used were social platforms (67.2%, 453/674) such as QQ, WeChat and Weibo, which can also provide the function of searching information except for the chat function, followed by traditional media digital platforms (62.0%, 418/674) such as People’s Daily and CCTV News and social news apps such as Headlines Today (40.5%, 273/674). In terms of COVID-19-related online information topics, respondents were most concerned about prevention (84%, 566/674), symptoms and manifestations (77.9%, 525/674), treatment drugs (48.2%, 325/674), inspection methods (45.5%, 307/674) and diagnosis (38.3%, 258/674).

**TABLE 3:** Characteristics of COVID-19-related online information seeking behavior.

Characteristic	Values (*N* = 674)
**Frequency *n* (%)**	
Once a week or less	52 (7.7)
2–6 times a week	116 (17.2)
1–3 times a day	335 (49.7)
4–6 times a day	77 (11.4)
6 times a day or above	94 (13.9)
**Duration *n* (%)**	
Less than 10 min	200 (29.7)
10–30 min	326 (48.4)
30–60 min	93 (13.8)
More than 1 h	55 (8.1)
**Information channels *n* (%)**	
Social platforms such as QQ, WeChat, and Weibo	453 (67.2)
Traditional media digital platforms such as People’s Daily and CCTV News	418 (62.0)
Social news apps such as Headlines Today	273 (40.5)
Short video platforms such as Tik Tok	251 (37.2)
General search engines such as Baidu	160 (23.7)
Q&A platforms such as Quora	58 (8.6)
Professional medical communication platform such as DXY	41 (6.1)
**Topics *n* (%)**	
How to prevent	566 (84.0)
Symptoms and manifestations	525 (77.9)
Treatment drugs	325 (48.2)
Inspection methods	307 (45.5)
How to diagnose	258 (38.3)
Efficacy and prognosis	201 (29.8)
Health service	188 (27.9)

### Comparison of Different Groups of Demographics and COVID-19-Related Online Information Seeking Behavior on Cyberchondria

As depicted in Table 4, gender (*P* = 0.023), age (*P* = 0.004), monthly income (*P* = 0.012) and education level (*P* = 0.017) were significantly associated with the C-CSS-12 total score. Males scored slightly higher than females (males 31.82 ± 12.27, females 29.75 ± 10.86), and the 20-to 30-year-old age group had the highest score (32.08 ± 11.18). The C-CSS-12 total score of the group with monthly income above CNY 8,000 (35.22 ± 12.75) was higher than other groups, and the C-CSS-12 total score of the group with a master’s degree and above (32.16 ± 11.68) was higher than other groups. The C-CSS-12 total score of the group with HP infection was higher than that of the group without HP infection (*P* = 0.013). The score of the C-CSS-12 subscale “Distress” with relatives suffering from chronic bronchitis was higher than that of the group without relatives with chronic bronchitis (*P* = 0.013). However, no significant relationship was found between vocation and the C-CSS-12 total score (*P* = 0.089).

**TABLE 4:** Cyberchondria (C-CSS-12) according to sociodemographic factors and COVID-19-related online information seeking behavior.

Variable	C-CSS-12/C-CSS-12 subscale score mean (SD)		*t*/F**	*P-*value
**Gender**			2.276	0.023*
Male		31.82 (12.27)		
Female		29.75 (10.86)		
**Age (years)**			3.950	0.004**
<20		28.84 (11.05)		
20–30		32.08 (11.18)		
31–40		31.95 (11.34)		
41–50		29.25 (11.74)		
>50		27.00 (12.81)		
**Education level**			4.120	0.017*
≤High middle school		28.94 (11.13)		
Undergraduate		31.48 (11.66)		
≥Postgraduate		32.16 (11.68)		
**Monthly income (CNY)**			2.961	0.012*
<2000		28.67 (10.52)		
2000–3000		30.72 (11.79)		
3001–4500		32.09 (11.75)		
4501–6000		31.01 (11.21)		
6001–8000		28.96 (12.73)		
>8000		35.22 (12.75)		
**Personal illness**			2.482	0.013*
Helicobacter pylori infection	Have 28	35.93 (12.88)		
	Not have (646)	30.42 (11.43)		
**Relative’s illness**			2.491	0.013*
Chronic bronchitis	Have 42	8.29 (3.20)^a^		
	Not have (632)	6.94 (3.42)^a^		
**Frequency**			4.805	0.001**
Once a week or less		26.29 (11.05)		
2–6 times a week		29.14 (11.29)		
1–3 times a day		30.49 (10.96)		
4–6 times a day		33.66 (11.52)		
6 times a day or above		33.04 (13.09)		
**Duration**			5.118	0.002**
Less than 10 min		28.18 (10.37)		
10–30 min		31.32 (11.90)		
30–60 min		31.86 (11.23)		
More than 1 h		33.65 (12.48)		
**Information channels**				
Traditional media digital platforms such as People’s Daily and CCTV News	Use (418)	6.76 (3.36)^a^	−2.497	0.013*
	Not use (256)	7.44 (3.46)^a^		
Social platforms such as QQ, WeChat and Weibo	Use (453)	9.13 (3.22)^b^	2.799	0.005**
	Not use (221)	8.39 (3.27)^b^		
Social news apps such as Headlines Today	Use (273)	31.73 (11.54)	1.997	0.046*
	Not use (401)	29.92 (11.48)		
Q&A platforms such as Quora	Use (58)	9.79 (3.50)^b^	2.221	0.027*
	Not use (616)	8.80 (3.22)^b^		
General search engines such as Baidu	Use (160)	32.28 (11.18)	2.050	0.041*
	Not use (514)	30.15 (11.60)		
**Topics**				
Treatment drugs	Search (325)	31.98 (11.56)	2.896	0.004**
	Not search (349)	29.42 (11.38)		
Inspection methods	Search (307)	31.80 (12.00)	2.380	0.018*
	Not search (367)	29.69 (11.05)		
How to diagnose	Search (258)	33.42 (12.12)	4.866	<0.001**
	Not search (416)	28.94 (10.81)		
Efficacy and prognosis	Search (201)	33.71 (12.17)	4.547	<0.001**
	Not search (473)	29.36 (11.01)		
Health service	Search (188)	32.69 (11.93)	2.870	0.004**
	Not search (486)	29.86 (11.29)		

*Statistically significant (*P* < 0.05). **Statistically significant (*P* < 0.01). ^*a*^Represents the C-CSS-12 subscale “Distress.” ^*b*^Represents the C-CSS-12 subscale “Compulsion”.

As for COVID-19-related online information seeking behavior, frequency (*P* = 0.001) and online duration (*P* = 0.002) were significantly associated with the C-CSS-12 total score; the C-CSS-12 total score of the group that searched 4–6 times a day (33.66 ± 11.52) was higher than that of the other groups. The C-CSS-12 total score of the group that searched online more than 1 h each time (33.65 ± 12.48) was highest among all groups. In terms of different information channels, the use of social news apps such as Headlines Today (*P* = 0.046) and general search engines such as Baidu (*P* = 0.041) were significantly associated with the C-CSS-12 total score, the use of traditional media digital platforms such as CCTV News was significantly associated with the score of the C-CSS-12 subscale “Distress” (*P* = 0.013), and the use of social platforms such as QQ (*P* = 0.005) and Q&A platforms such as Quora (*P* = 0.027) were significantly associated with the score of the C-CSS-12 subscale “Compulsion.” With regard to searching COVID-19-related information topics, treatment drugs (*P* = 0.004), inspection methods (*P* = 0.018), diagnosis (*P* < 0.001), efficacy and prognosis (*P* < 0.001), and health services (*P* = 0.004) were significantly associated with the C-CSS-12 total score.

### Relationship Between Cyberchondria and Other Measured Variables

As depicted in Table 5, the average of the SHAI total score was 18.23 ± 11.09, while that of the eHEALS total score was 30.72 ± 6.54 and that of the PHQ-15 was 4.01 ± 4.95.

**TABLE 5:** Spearman’s correlations among C-CSS-12, SHAI, eHEALS, PHQ-15 (*n* = 674).

Correlations*	Score mean (SD)	C-CSS-12 total	C-CSS-12 excessiveness	C-CSS-12 compulsion	C-CSS-12 distress	C-CSS-12 reassurance seeking	eHEALS total	SHAI total	SHAI IK	SHAI NC	PHQ-15 total
C-CSS-12 total	30.65 (11.53)	1									
C-CSS-12 excessiveness	8.89 (3.25)	0.848**	1								
C-CSS-12 compulsion	6.80 (3.23)	0.912**	0.659**	1							
C-CSS-12 distress	7.02 (3.42)	0.889**	0.638**	0.841**	1						
C-CSS-12 reassurance seeking	7.94 (3.27)	0.855**	0.680**	0.696**	0.625**	1					
eHEALS total	30.72 (6.54)	0.115**	0.169**	0.062	0.001	0.177**	1				
SHAI total	18.23 (11.09)	0.623**	0.523**	0.573**	0.591**	0.496**	0.027	1			
SHAI IK	14.00 (8.71)	0.625**	0.527**	0.569**	0.595**	0.497**	0.031	0.985**	1		
SHAI NC	4.23 (2.92)	0.504**	0.416**	0.478**	0.469**	0.402**	0.010	0.858**	0.757**	1	
PHQ-15 total	4.01 (4.95)	0.290**	0.244**	0.260**	0.303**	0.209**	−0.041	0.438**	0.438**	0.355**	1

*Statistically significant (*P* < 0.05). **Statistically significant (*P* < 0.01).

Besides, Table 5 shows the relationships among cyberchondria (C-CSS-12), health anxiety (SHAI), e-health literacy (eHEALS) and Patient Health Questionnaire-15 (PHQ-15). Cyberchondria showed positive relationships with health anxiety (*r* = 0.623, *P* < 0.01), e-health literacy (*r* = 0.115, *P* < 0.01), and physical symptoms (*r* = 0.290, *P* < 0.01). Health anxiety correlated positively with the physical symptoms (*r* = 0.438, *P* < 0.01).

### Multiple Linear Regression Model for Cyberchondria

According to the results of the *t*-test or analysis of variance, we included gender, age, education level, monthly income, personal illness, relative illness, frequency, duration, topics and information channels in the multiple linear regression model. Table 6 presents the multiple regression results on the associations of sociodemographics, personal/relatives’ illness, COVID-19-related online information seeking behavior characteristics, eHEALS, the SHAI, and the PHQ-15 with the C-CSS-12 total score. The eHEALS score (β = 0.162 > 0, *P* = 0.003), SHAI total score (β = 0.598 > 0, *P* < 0.001), use of general search engines such as Baidu (β = 1.867 > 0, *P* = 0.039), and searching for information on how to diagnose COVID-19 (β = 2.280 > 0, *P* = 0.020) were independent risk factors for the impact of the C-CSS-12 total score, while searching lasting less than 10 min each time (β = −2.992 < 0, *P* = 0.048), using traditional media digital platforms such as People’s Daily and CCTV News (β = −1.650 < 0, *P* = 0.024), and using professional medical communication platforms such as DXY (β = −4.189 < 0, *P* = 0.007) were independent protective factors for the impact of the C-CSS-12 total score.

**TABLE 6:** The multiple linear regression results on associated factors with cyberchondria.

Model		Unstandardized coefficients		Standardized coefficients	*t*	*P*	Collinearity statistics	
		β	Std. error	β			Tolerance	B
(Constant)		15.163	3.125		4.852	<0.001**		
eHEALS score		0.162	0.055	0.092	2.964	0.003**	0.890	1.123
SHAI total		0.598	0.036	0.575	16.725	<0.001**	0.726	1.378
Duration	Less than 10 min	–2.992	1.510	−0.119	−1.981	0.048*	0.239	4.176
	10–30 min	–1.594	1.412	−0.069	−1.129	0.259	0.229	4.367
	30–60 min	–1.679	1.577	−0.050	−1.064	0.288	0.385	2.597
Information channel	Traditional media digital platforms such as People’s Daily and CCTV News	–1.650	0.727	−0.070	−2.270	0.024*	0.916	1.092
	Social platforms such as QQ, WeChat and Weibo	–0.516	0.774	−0.021	−0.667	0.505	0.864	1.157
	Social news apps such as Headlines Today	0.538	0.761	0.023	0.706	0.480	0.816	1.225
	Short video platforms such as Tik Tok	0.688	0.759	0.029	0.906	0.365	0.846	1.182
	Q&A platforms such as Quora	–0.356	1.350	−0.009	−0.263	0.792	0.795	1.257
	General search engines such as Baidu	1.867	0.902	0.069	2.070	0.039*	0.774	1.292
	Professional medical communication platform such as DXY	–4.189	1.538	−0.087	−2.723	0.007**	0.843	1.186
Topics	Inspection methods	–0.344	0.867	−0.015	−0.397	0.691	0.612	1.635
	How to diagnose	2.280	0.976	0.096	2.337	0.020*	0.507	1.974
	Treatment drugs	–0.470	0.835	−0.020	−0.563	0.574	0.654	1.528
	Treatment drugs	1.223	1.021	0.049	1.198	0.232	0.523	1.914
	Health service	–0.936	0.906	−0.036	−1.033	0.302	0.691	1.448
	*R* ^2^					0.453		
	*F*					14.666		
	*P*					<0.001**		
	Dependent variable: C-CSS-12 total														

*Statistically significant (*P* < 0.05), **Statistically significant (*P* < 0.01).

*Predictors (constant): eHEALS score; SHAI total; PHQ-15 total; gender; age; monthly income; education level; frequency; duration; personal illness on helicobacter pylori infection; relatives’ illness on chronic bronchitis; information channels on traditional media digital platforms, social platforms, social news apps, short video platforms, Q&A platforms, general search engines, professional medical communication platform; topics on inspection methods, how to diagnose, treatment drugs, treatment drugs, health service.*

## Discussion

This study was a cross-sectional, anonymous, self-report questionnaire survey that aimed to investigate the status of cyberchondria as well as its influencing factors during the virus epidemic in community residents in Nanyang city, China.

In our study, we found that the average C-CSS-12 total score of residents was 30.65 ± 11.53 during the virus epidemic; 25% of participants scored 22 or below, 50% scored 23 to 38, and 21.9% scored 39 to 60. Gender, age, monthly income, education level, personal illness with HP infection, relatives’ illness with chronic bronchitis, COVID-19-related online information seeking frequency and duration were all significantly associated with cyberchondria. Health anxiety, the use of general search engines and searching for information on how to diagnose COVID-19 were independent risk factors for cyberchondria, while searching lasting less than 10 min each, the use of traditional media digital platforms and professional medical communication platforms were independent protective factors.

### Status of Cyberchondria

Overall, the results should be interpreted against the background of the situation in China at the time the study was conducted. The study took place from February 1 to 15, 2020,at the peak of the virus outbreak in China. According to the literature, only a small amount of data on the level of cyberchondria were present, especially for the general population (Vismara et al., 2020). In our study, the average C-CSS-12 total score of residents was 30.65 ± 11.53 and 21.9% of participants scored 39 and above, which was higher than the score of German residents during the epidemic (22.45 ± 7.28) (Jungmann and Witthoft, 2020) and much higher than the baseline score (Vismara et al., 2020). Another study found that Chinese residents had a C-CSS-12 total score of 42.50 ± 6.01 under the epidemic (Zheng et al., 2021). At that time in China, the large number of patients with few medical supplies, the various transmission route of the virus, the uncertainty of the incubation period, and possible asymptomatic infection increased the anxiety and stress of residents (Yang et al., 2020). In particular, unprecedented lockdown measures and social isolation made it possible for people to search for COVID-19-related information more frequently online (Chinese Center for Disease Control and Prevention, 2020). Besides, the difficulty of dealing with uncertainty, information overload, the dubious credibility of online information and the failure to seek reassurance online have made some residents more anxious, which might be the reasons for the increase in the severity of cyberchondria.

### Sociodemographic and Health Condition Factor Distribution in Cyberchondria

We found that the C-CSS-12 total score of men during the epidemic was slightly higher than that of women, which was consistent with some studies (Akhtar and Fatima, 2020). This difference was especially reflected in the C-CSS-12 subscales “Compulsion” and “Reassurance Seeking.” The 20- to 30-year-old age group had the highest C-CSS-12 total score, which could be explained by the likelihood of younger adults using the internet more than older adults and was also consistent with the results of Doherty-Torstrick (Doherty-Torstrick et al., 2016) and Bajcar (Bajcar et al., 2019). Some studies have noted that due to the absence of a consensus definition, data reliably linking cyberchondria with sociodemographic variables, including gender and age, are conflicting and scarce (Vismara et al., 2020). Barke reported that age was unrelated to the CSS total, and women had a higher CSS score than men (Barke et al., 2016). Bajcar found no effect of gender but a significant negative effect of age on CSS scores (Bajcar et al., 2019). Another investigation conducted among university students reported higher male scores for the subscale “Compulsion” than female scores, with no gender difference in the total CSS score (Bati et al., 2018). In this study, residents with a monthly income of more than CNY 8000 and a master’s degree or above had higher C-CSS-12 total scores, which might be related to their greater health concerns and more in-depth health information search demands. In addition, the score of the C-CSS-12 subscale “Distress” among those with relatives suffering from chronic bronchitis was higher than that of the group without, which might be related to the concern of these residents that the underlying respiratory diseases of their relatives may increase their relatives’ susceptibility to new coronary pneumonia or increase the possibility of illness in the family. However, the results of multiple linear regression indicated that gender, age, monthly income, education level, personal illness with HP infection, and relatives’ illness with chronic bronchitis were not independent influencing factors for cyberchondria.

### Association With Health Anxiety

This study was designed to determine the general anxiety and cyberchondria of residents affected by the epidemic rather than the specific ones on COVID-19. We found that cyberchondria had a strong positive correlation with health anxiety (*r* = 0.623); the regression coefficient was 0.596, which is consistent with most literature results (McMullan et al., 2019). Several studies have found that residents with elevated health anxiety experience greater anxiety during and after online health searches and report more frequent and longer searches compared to those with lower or normal levels of health anxiety (Doherty-Torstrick et al., 2016; Singh and Brown, 2016; Te Poel et al., 2016). And in the context of the pandemic, recent studies showed that some individuals exposed to social media and incorrect information about COVID-19 perceived anxiety and threat more strongly (Kavakli et al., 2020). The higher the current virus anxiety, the stronger the cyberchondria (Jungmann and Witthoft, 2020). The average SHAI total was higher than the anxiety level in Germany during the epidemic (Jungmann and Witthoft, 2020), and the average C-CSS-12 total score was higher than that in Germany as well. This might be related to Chinese residents’ awareness of the disease, concerns about the prevention and control of the epidemic, the degree of attention to epidemic information, and the ability to distinguish between credible and non-credible sources of online information (Chang et al., 2020; Yang, 2021). In another study, cyberchondria was reported as a risk factor for “coronavirus anxiety,” which was reduced with full understanding knowledge of the pandemic (Jungmann and Witthoft, 2020). Accordingly, this paper further confirmed the positive relationship between health anxiety and cyberchondria.

### Association With COVID-19-Related Online Information Seeking Behavior

Our results indicated that COVID-19-related online information seeking frequency and online duration were significantly associated with the C-CSS-12 total score. The higher the frequency and the longer the time, the higher the score. Some studies have reported that illness-related information consumption could cause worry or anxiety about one’s health (Liu, 2020), and more frequent social media exposure to COVID-19 is positively correlated with anxiety symptoms (Elhai et al., 2020; Gao et al., 2020). This might be related to the fact that during long-term attention to health information on the epidemic, excessive stimulation can inactivate the happiness produced by the brain’s secretion of dopamine, lower the excitement threshold, cause emotional disorders, and weaken emotional regulation and processing capabilities, which may cause negative emotions such as anxiety (Gao et al., 2020). Some studies have suggested that patients with “moderate-severe health anxiety” should avoid using the internet for health-related information based on a strong association between health anxiety and cyberchondria (Doherty-Torstrick et al., 2016). However, it may be difficult for people to do this in the digital age. The results of the multiple linear regression indicated that searching for less than 10 min each time was an independent protective factor for cyberchondria, which might be a suggested and recommended approach. Searching for COVID-19-related online information topics, treatment drugs, inspection methods, diagnostic methods, efficacy and prognosis, and health services was significantly associated with the C-CSS-12 total score; in particular, searching for information on diagnostic methods was an independent risk factor. This may be explained by the fact that people might notice similar symptoms and thus seek information for self-diagnosis given the high infectivity of the virus, thus increasing their concerns (Buhr and Dugas, 2009). Additionally, to a certain extent, the findings reflect that online information on the diagnosis of new coronary pneumonia was possibly unclear and conflicting at the time, and determining whom to trust became a guessing game. The internet is not designed to always provide relevant, accurate, non-conflicting, non-ambiguous or reassuring information, and misinformation on COVID-19 has been proliferating on the internet. These make it difficult to distinguish between reliable and unreliable information and leads to failure to obtain the expected reassurance in the course of online health-related searches, which increases health anxiety (Starcevic, 2017).

Our research also showed that Chinese residents tend to obtain information about the epidemic through traditional media digital platforms, social platforms, news apps, short video platforms, and general search engines. However, due to widespread access to the enormous amount of information facilitated by various digital media platforms, individuals might be overwhelmed with uncertainty, and their concerns regarding the pandemic may be increased. We found that using general search engines such as Baidu and Sougou was an independent risk factor for cyberchondria (β = 1.867). This may be related to the multisource information, high degree of openness, poor consistency, and large differences in information accuracy and completeness of this type of search engine, which make it more difficult for people to distinguish whether the information is reliable. In addition, when searching online, users are more inclined to view and click on titles containing potentially dangerous medical terms (White and Horvitz, 2013), which may also be related to the escalation of anxiety. Starcevic V (Starcevic, 2017) proposed improving the presentation of online health information and online health-related engine search results to present online health information in a way that is clear and user-friendly. Furthermore, checking online search results based on the true probability of the relationship between specific symptoms and diagnosis methods such as ranking may reduce misunderstandings and the escalation of health anxiety. We also found that using traditional media digital platforms (β = −1.650), such as the People’s Daily and CCTV News, was an independent protective factor for cyberchondria, which may be related to the supervision of the government and relevant departments for information release through these channels. The authority and quality of the information is better, which could give users more comfort when seeking COVID-19-related health information. Likewise, information from professional medical communication platforms such as DXY is mostly professionally certified by the doctor’s editorial department. This channel is becoming increasingly popular with residents and could provide relatively reliable medical content and advice (Venkatasubramanian, 2020). Therefore, it was suggested that the choice of information channels, especially the network information quality of platforms, may have an impact on cyberchondria, and that improving the information quality of different channels or guiding users to choose authoritative platforms may be good interventions for cyberchondria.

### Association With E-Health Literacy and Physical Symptoms

Some scholars have noted that cyberchondria is considered a specific form of health-related problematic internet use (Vismara et al., 2020), especially involving the ability to distinguish reliable information (Starcevic, 2017). It is shown that e-health literacy could negatively moderate the indirect effect of affective responses on cyberchondria (Zheng et al., 2020). Some studies have noted that people with high e-health literacy are able to understand the information that they find on the internet, verify the veracity of the information, and use this information to promote health behaviors (Huang et al., 2020). They might avoid excessive online health searches, although they are anxious about their health status (Zheng et al., 2020). However, unexpectedly, we found a slightly positive correlation between e-health literacy and cyberchondria in our study. This might be that e-health literacy was positively correlated with online time and frequency (Yuan et al., 2015; Wong and Cheung, 2019) to the same extent as cyberchondria and served as an enabler to online health information seeking (Li et al., 2014). Therefore, the impact of e-health literacy on cyberchondria needs further exploration.

In addition, cyberchondria was slightly positively correlated with personal physical symptoms, indicating that cyberchondria is a comprehensive problem involving psychology, physiology, online information search behavior, information resources, and social public health. Recent study found that the psychosomatic symptom level was positively related to perceived COVID-19 threat and anxiety (Gica et al., 2020; Ran et al., 2020; Xu et al., 2020). In particular, individuals with symptoms, such as pain catastrophizing (Gibler et al., 2019), searched online for more information about their symptoms, resulting in disruption of daily functioning, escalations in health-related concerns, excessive health-related checking behaviors and greater healthcare utilization.

## Limitations

The research reported in our paper has several limitations. Since cyberchondria is a relatively new area of research, there is no diagnostic criteria yet, and the CSS-12 does not yet define the diagnostic cut-off score, so we cannot scientifically distinguish the severity level and just describe the distribution of scores. How to classify the severity will be the research direction in the future. Second, the cross-sectional design of the study did not allow exploration of causal or temporal relationships between variables. A longitudinal design would better assist in establishing that link. In addition, we used a sample from only one large city. To understand the overall status of cyberchondria in China, the sample size needs to be further expanded.

## Conclusion

Our findings showed that nearly a quarter of the participants scored 39 or higher on the C-CSS-12 in Nanyang city during the pandemic, which should be taken seriously. Health anxiety and COVID-19-related online information seeking behavior including online duration, topics and choice on different information channels were important influencing factors of cyberchondria. These findings have implications for further research and clinical practice on cyberchondria in China.

## Data Availability Statement

The raw data supporting the conclusions of this article will be made available by the authors, without undue reservation.

## Ethics Statement

The studies involving human participants were reviewed and approved by the institutional review board (IRB) of the Third Xiangya Hospital, Central South University. The patients/participants provided their written informed consent to participate in this study. Written informed consent was obtained from the individual(s) for the publication of any potentially identifiable images or data included in this article.

## Author Contributions

X-QP conceptualized and designed the study as well as the investigation. She also drafted the original manuscript. YC designed the questionnaires together with X-QP, and distributed questionnaires with Y-CZ, FL as well as H-YH. X-QP and YC carried out the initial analyses and interpreted the data and review and revised the manuscript. Y-CZ, FL, and H-YH distributed and collected questionnaires together with YC. TL and P-PD reviewed the initial data and analytical outcome. A-JL and W-ZX obtained funding for the research and supervised the procedure of the whole investigation evaluated the project. All authors contributed to the article and approved the submitted version.

## Conflict of Interest

The authors declare that the research was conducted in the absence of any commercial or financial relationships that could be construed as a potential conflict of interest.

## Publisher’s Note

All claims expressed in this article are solely those of the authors and do not necessarily represent those of their affiliated organizations, or those of the publisher, the editors and the reviewers. Any product that may be evaluated in this article, or claim that may be made by its manufacturer, is not guaranteed or endorsed by the publisher.

## Footnotes

1
http://www.wjx.cn

## Abbreviations

C-CSS-12: The Chinese Short Form of the Cyberchondria Severity Scale
SHAI: Short Health Anxiety Inventory
eHEALS: eHealth Literacy Scale
PHQ-15: Patient Health Questionnaire-15
HP: helicobacter pylori.
